# *Pseudomonas fluorescens* BsEB-1: an endophytic bacterium isolated from the root of *Bletilla striata* that can promote its growth

**DOI:** 10.1080/15592324.2022.2100626

**Published:** 2022-08-03

**Authors:** Yuanshuang Wu, Suhui Xiao, Jiaseng Qi, Yongchang Gong, Kunzhi Li

**Affiliations:** Faculty of Life Science and Technology, Kunming University of Science and Technology, Kunming, PR China

**Keywords:** Pseudomonas fluorescens, growth-promoting activity, endophytic colonization, rooting and transplant survival rate, the expansion and growth of *Bletilla striata* tuber

## Abstract

An endophytic *Pseudomonas fluorescens* (BsEB-1) was obtained from the roots of *Bletilla striata*. We investigated its growth-promoting properties and observed the impact of its inoculation on both the growth and polysaccharide content of *Bletilla striata* tubers. It was found that BsEB-1 possessed three growth-promoting activities: phosphate-solubilizing, produced indoleacetic acid (IAA) and siderophores, but had no nitrogen-fixing activity. BsEB-1 could rapidly attach to the root hairs of *Bletilla striata* tissue culture seedlings and endophytically colonize the region of maturation in the roots. It also significantly promoted the rooting and transplant survival rate of the seedlings, as well as the growth and expansion of the tubers, but did not increase their polysaccharide content. *Pseudomonas fluorescens* BsEB-1 exhibits potential for applications in the artificial planting of *Bletilla striata.*

## Introduction

The plant rhizosphere is comprised of the specific areas (2–3 mm) of the soil that exist in immediate proximity to plant root systems. It contains a wide variety and dense population of microorganisms that can either promote or inhibit the growth of plants.^1^ Endophytic bacteria are considered a subclass of rhizospheric bacteria, commonly called as plant growth promoting rhizobacteria (PGPR). At present, a variety of (PGPR) have been identified from various soils, and these mainly include *Bacillus* and *Pseudomonas*.^2^ Plant growth-promoting rhizobacteria include soil bacteria that can colonize the surface of the root system (and sometimes the root inner tissues) to stimulate the growth and health of the plant. Among them, *Pseudomonas fluorescens* occupies an absolute advantage in the rhizosphere of many plants.^3^

The earliest research conducted on *Pseudomonas fluorescens* began in 1978. It found that the yield of potatoes increased after the crop was treated with *Pseudomonas fluorescens*. Some studies have found that *Pseudomonas fluorescens* can regulate the plant defense response through plant hormone signaling pathways, such as those involving salicylic acid (SA),^4^ or through some metabolites, for example, the polyketide antibiotic 2,4-diacetylphloroglucinol (2,4-DAPG),^5^ among others. Many secondary metabolites produced by *Pseudomonas fluorescens*; such as phenazines, 2,4-diacetylphloroglucinol, pyoluteorin, pyrrolnitrin, lipopeptides, and hydrogen cyanide;^6^ have shown great potential in the control of plant diseases.^7^ It has been found that siderophores produced by *Pseudomonas fluorescens* could inhibit a variety of plant diseases and promote plant growth.^8,9^ Several studies have found that *Pseudomonas fluorescens* could colonize the root inner tissues to stimulate the growth and health of the host plant.^10^ *Pseudomonas fluorescens* mainly exhibits its plant-regulating functions from the following three aspects: plant root colonization, the induction of systemic plant defense mechanisms, and the production of secondary metabolites that exhibit antibacterial activities.

*Pseudomonas fluorescens* may have co-evolved with plants as endophytic bacteria. In such a relationship, the host plants would have provided a stable environment and abundant photosynthetic products for the resident entophytes, providing the materials and energy to be consumed to support microbial growth *in vivo*, while the endophytes promoted the growth of the host plant and improved its resistance to diseases, thereby enhancing its survival and competitiveness in the ecosystem.^11,12^ In interacting with medicinal plants, endophytes not only promote the primary metabolism of hosts, but also enhance the accumulation of secondary metabolites that support plant defense systems by triggering corresponding responses of the host.^13,14^ A variety of plants are known to be hosts to endophytic bacteria, many of which belong to the genus *Pseudomonas*.^15^ In *Atractylodes lancea, Pseudomonas fluorescens* has been shown to promote the production of plant photosynthetic products and increase the accumulation of secondary metabolites such as sesquiterpenes, but was not observed to increase plant biomass.^16^ Taken together, these studies indicate that *Pseudomonas fluorescens* is genetically diverse and performs synergistic effects with plants and the surrounding environment. Environmental conditions were found to exert a considerable impact on the development and genetic differentiation of *Pseudomonas fluorescens*.^17^ Because of this, the functions of *Pseudomonas fluorescens* differ between different plants and environments.

*Bletilla striata* is a component of traditional Chinese medicine in China, often incorporated from its dried tubers. It has been widely used in the treatment of hemoptysis and traumatic bleeding, as well as in treating chapped skin, swelling, and ulcer bleeding.^18^ It has also commonly been used as a raw material for both drug carrier and film-forming substances.^19,20^ With such diverse applications, *Bletilla striata* possess high medicinal and commercial value. Unfortunately, the over-exploitation of the wild resources of *Bletilla striata* combined with its low reproduction rate under natural conditions have led to a steep decline in the species as well as the loss of its genetic diversity, ultimately degrading its quality.^21^ At present, the production of *Bletilla striata* primarily depends on artificial planting, but the medicinal quality of artificially planted medicinal materials is often lower than that of wild medicinal materials. Strategies to improve the yield and medicinal qualities of artificially planted *Bletilla striata* are therefore needed.

Research on the diversity of the endophytic bacteria that colonize *Bletilla striata* has identified the *Pseudomonas* genus,^22^ the endophytic bacteria residing within plant cells might alter the content of *Bletilla striata* polysaccharide (BSP),^23^ but their effect on the growth and metabolism of *Bletilla striata* has remained unknown. In this paper, we isolated the endophytic *Pseudomonas fluorescens* from the root of *Bletilla striata* and investigated its effect on the growth of the host plant. The growth-promoting effects and transplant survival rates of *Bletilla striata* tissue culture seedlings were investigated, as well as the content of *Bletilla striata* polysaccharide (BSP). The results provide a basis for the follow-up study of the interaction between *Pseudomonas fluorescens* and *Bletilla striata*, thereby contributing to the improved artificial planting of *Bletilla striata*.

### Materials and methods

#### Plant material and bacteria

The *Bletilla striata* tissue culture seedlings were provided by Kunming Yingwu Agricultural Technology Ltd. *Bletilla striata* seedlings were collected from the planting base of Kunming Yingwu Agricultural Technology Ltd. located in Mile Yunnan. The seedling growth medium used was comprised of MS + 2.5 mg/L 6-BA + 1.0 mg/L NAA and the rooting medium used was comprised of 1/2 MS + 1.5 mg/L KT + 0.5 mg/L NAA, all with 10 g/L agar, 20 g/L sucrose, pH 5.6–5.8.

The bacterium *Pseudomonas fluorescens* was separated from the roots of planted *Bletilla striata* seedlings. The detailed process of separation was as follows: after washing and surface disinfecting the roots of fresh *Bletilla striata* with 75% ethanol for 1 min and then 2% NaClO for 6 min, the roots were cut and put into sterile water with shaking for 20 min. The solution was serially diluted by 10X and then applied on LB medium. The monoclone was picked out and purified by streak plate method. The purified clones were cultured on King’s B medium (20 g/L peptone, 1.5 g/L K_2_HPO_4_, 1.5 g/L MgSO_4_, 15 g/L Agar, pH 7.2 ± 0.2) and fluorescence was subsequently observed under the UV transilluminator of the gel imaging system each day. Bacteria that produced yellow fluorescence within 5 days were considered candidates of *Pseudomonas fluorescens*.

#### Molecular identification and phylogenetic analysis of endogenous Pseudomonas fluorescens

The 16S rRNA of the purified cultures of bacteria was sequenced by Kunming Tsingke Biotechnology Limited Company. Universal primers for 16S were used for PCR amplification and the identification of bacteria.^24^ The primer sequence used was:

27F: 5ʹAGAGTTTGATCCTGGCTCAG3’;

1429R:5ʹGGTTACCTTGTTACGACTT 3’.

The obtained partial sequence of 16S rRNA was compared in the GenBank database using the BLASTN tool (blast.ncbi.nlm.nih.gov/Blast.cgi) and a phylogenetic tree was constructed by the use of MEGA X software (version 6.06).

#### Analysis of the growth-promoting activity of Pseudomonas fluorescens

##### Nitrogen-fixing activity

The bacteria were washed with sterile water 3X and then resuspended in water. A volume of 20–50 μL of bacterial suspension was evenly coated on nitrogen-free solid Ashby’S Medium (mannitol, 10 g/L; KH_2_PO_4_, 0.2 g/L; MgSO_4_.7H_2_O, 0.2 g/L; NaCl, 0.2 g/L; CaSO_4_.2H_2_O, 0.1 g; CaCO_3_, 5 g/L; Agar, 18 g/L, pH6.8–7.2) and cultured at 28°C for 2–7 days.

##### Phosphate-solubilizing activity

Bacterial strains were washed with sterile water 3X and then resuspended in water. A volume of 20–50 μL of bacterial suspension was evenly coated on NBRIP solid medium (glucose, 10 g/L; Ca_3_(PO_4_)_2_, 5 g/L; MgCl_2_.6H_2_O, 5 g/L; MgSO_4_.7H_2_O, 0.25 g/L; KCl, 0.2 g/L; (NH_4_)_2_SO_4_, 0.1 g/L; Agar, 18 g/L) and then incubated at 28°C for 7 days. The observation of a clear halo having formed around the colonies was deemed to have indicated phosphate-solubilizing activity.

The soluble phosphorus in the bacterial solution was detected by the molybdenum antimony resistance colorimetric method. Briefly, 20 μL of bacterial solution that had been resuspended with sterile water was added to 40 ml of NBRIP liquid medium and then cultured at 28°C, 150 rpm for 4 days. The bacterial solution was then centrifuged at 8000 rpm for 10 min, after which 1 ml supernatant was taken into a 50 ml volumetric flask, then 5 ml of molybdenum antimony resistance chromogenic agent was added, and the volumetric flask was filled to 50 ml with distilled water. After standing for 30 min, the absorbance of the mixture at OD_660_ was measured. The NBRIP liquid medium without bacteria was taken as the blank control. Potassium dihydrogen phosphate was used as a soluble phosphorus standard to make the standard curve, and the experimental content of soluble phosphorus was calculated using this curve.

##### Indoleacetic acid (IAA) production

The bacteria were inoculated into LB liquid medium either containing L-tryptophan (200 mg/L) or without L-tryptophan. After culturing at 28°C and 150 rpm for 48 h, the cultures were centrifuged at 10000 rpm for 10 min. The supernatant was added with an equal volume of Salkowski’s colorimetric solution (4.5 g of FeCl_3_ per liter in 10.8 M H_2_SO_4_) and stood in the dark for 30 min, then the OD_530_ was measured. The content of IAA was calculated according to the standard curve of IAA.

##### Siderophore production

*Pseudomonas fluorescens* was inoculated in King’s B liquid medium for several days. When bright fluorescence was generated, the bacterial solution was centrifuged at 5000 rpm for 5 min, and the OD_400_ of the supernatant was detected.

#### Transformation of Pseudomonas fluorescens with the mRFP1 gene

##### Construction of a prokaryotic expression vector carrying the mRFP1 gene

The red fluorescent protein gene *mRFP1* was obtained from the bacterial strain preserved in our laboratory. The primers for the *mRFP1* gene contained the restriction endonuclease sites of *Xho* I and *Eco*R I, respectively:

F: ctcgagATGGCCTCCTCCGAGGACGT

R: gaattcTTAGGCGCCGGTGGAGTGGC

The PCR products were purified with a Gel Extraction Kit (Takara), constructed into a PMD-18 T vector, and transformed into *E. coli* DH5α. The monoclone was sequenced and the correct one was amplified. The PMD-18 T-*mRFP1* and pBBR1MCS-2 prokaryotic expression vectors were extracted respectively, and the target fragment *mRFP1 gene* and the linear pBBR1MCS-2 vector were obtained by double enzyme digestion and gel extraction. The recombinant pBBR1MCS-2- *mRFP1* was generated through a ligation reaction using a DNA ligation kit (Takara).

##### Preparation of competent Pseudomonas fluorescens cells

The monoclonal bacteria were inoculated in LB liquid medium and cultured at 28°C, 150 rpm overnight, then 100 μL bacterial solution was transferred into 10 mL of fresh LB medium. When the OD_600_ reached ~0.6, the bacterial solution was placed in an ice bath for 30 min. The bacteria were collected by centrifugation and resuspended in 5 mL of precooled 0.025 mM CaCl_2_ for 2 hours in an ice bath. The bacteria were recollected by centrifugation and resuspended in 1 mL of precooled 0.025 mM CaCl_2_ for use.

##### Transformation of Pseudomonas fluorescens with the prokaryotic expression vector

A volume of 2 μL (~1 μg) of the pBBR1MCS-2- *mRFP1* vector was added to 100 μL of competent *Pseudomonas fluorescens* cells and mixed evenly. The mixture was heated to cause heat shock at 42°C for 3 min after being cooled in an ice bath for 30 min. The mixture was then placed in an ice bath for 5 min before 800 μL of LB liquid medium was added. The bacteria solution was centrifuged at 3000 rpm for 3 min after 3 h of culture. The precipitate was resuspended in 100 μL of LB liquid medium, then coated on LB solid medium which contained 100 μg/mL of kanamycin and cultured at 28°C for ~40 h. The monoclone was picked out and detected by PCR to determine the presence of the *mRFP1* gene. The expression of mRFP1 was confirmed by observation under a laser confocal microscope.

#### Effects of co-culturing: Pseudomonas fluorescens on the growth of Bletilla striata tissue culture seedlings

##### Observation of endophytic colonization by laser confocal microscope

When the MS solid medium cooled to ~50°C after sterilization, *Pseudomonas fluorescens* possessing the *mRFP1* gene was added to the medium and packed into the culture dishes after rapid mixing. The *Bletilla striata* tissue culture seedlings with roots attached were transplanted into MS solid medium. The roots of the B*letilla striata* seedlings were washed with EDTA solution and sliced longitudinally, and then the colonization of *Pseudomonas fluorescens* was observed under a laser confocal microscope every 3 days.

##### Effects of co-culturing with Pseudomonas fluorescens on the rooting of Bletilla striata tissue culture seedlings

The bacteria were cultured in LB liquid medium. When the OD_600_ of the bacterial solution reached 0.6–0.8, 5 ml of suspension was added to 500 ml of MS rooting solid medium at 50°C, quickly mixed, and then packed into a tissue culture bottle. Rootless seedlings of *Bletilla striata* with similar heights and sizes were inoculated into sterile MS rooting medium and then supplemented with either the bacteria or exogenous IAA. The growth of the *Bletilla striata* seedlings and roots was observed, and the rooting rate was determined after a month.

##### Effects of co-culturing with Pseudomonas fluorescens on the transplant survival rate of Bletilla striata tissue culture seedlings

*Bletilla striata* tissue culture seedlings were grouped by the same growth conditions after being cultured either with or without BsEB-1 and then transplanted into sterile soil for potting after seedling refining. The growth of tissue culture seedlings was observed and the survival rates were compared.

##### Determination of the polysaccharide content in Bletilla striata tubers

After being potted for two months, each tuber was measured for size, weight, and polysaccharide content. The tuber size was expressed by the long diameter, short diameter, and height of the bulb (mm). The polysaccharide content was determined by UV spectrophotometer with anthrone-H_2_SO_4_ colorimetry. Briefly, 0.2 g of tuber powder was extracted with 50 mL of simmering water by the heating reflux method for 1 h. Then, 5 mL of the solution was precipitated by 30 mL of absolute EtOH at 4°C for 1 h. The precipitate was then dissolved in water and added to 100 ml to form a crude polysaccharide solution. An amount of crude polysaccharide solution was diluted to 2 mL and then 3 mL of anthrone solution (0.2 g anthrone in 100 mL concentrated sulfuric acid) was added and the OD_625_ was detected. The polysaccharide content was calculated according to the standard curve, in which glucose was used as the standard.

#### Statistical analysis

Statistical data were expressed as the means ± standard deviation. Statistical analysis was performed by using Excel 2010. Comparisons between the different groups (either with or without bacteria) were determined by the T-test (P˂0.05). Some data were only used to indicate the activity of the bacteria themselves and expressed as a numerical number; this included data for the phosphate-solubilizing activity, IAA, and siderophore production.

## Results

### Isolation and identification of endophytic Pseudomonas fluorescens from Bletilla striata

**Figure 1.**
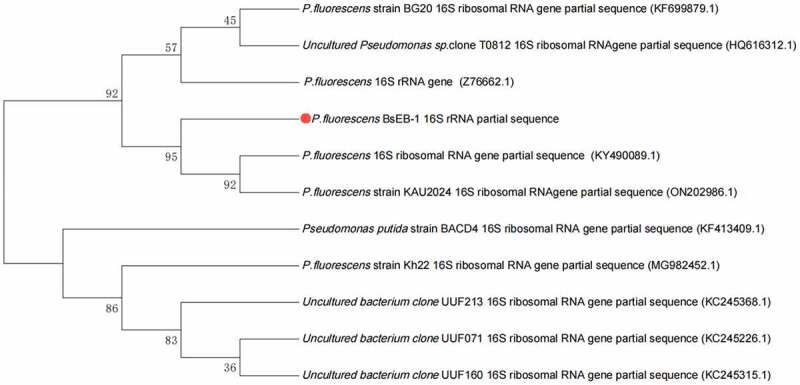
Phylogenetic tree of *Pseudomonas fluorescens* BsEB-1 16S rRNA sequence.

The 16S rRNA of *Pseudomonas fluorescens* extracted from *Bletilla striata* was sequenced (shown in SEQ1.txt in the supplied files). The results suggested that there was only one strain of *Pseudomonas fluorescens* present, which was named BsEB-1, and the strain has been kept in the China General Microbiological Culture Collection Center (CGMCC No. 23364). The 16S rRNA sequence of BsEB-1 was subject to the BLAST algorithm in NCBI (https://blast.ncbi.nlm.nih.gov/). The first 10 results are listed in Table 1 and the phylogenetic tree is shown in Figure 1.

**Table 1.** The result of sequence BLAST in NCBI of *Pseudomonas fluorescens* BsEB-1 16S rRNA.

Description	Scientific Name	Max Score	Total Score	Query Cover	E value	Per. Ident	Acc. Len	Accession
Pseudomonas fluorescens 16S ribosomal RNA gene, partial sequence	Pseudomonas fluorescens	1177	2197	99%	0	94.33	1509	KY490089.1
Pseudomonas fluorescens strain KAU2024 16S ribosomal RNA gene, partial sequence	Pseudomonas fluorescens	1177	2197	99%	0	94.33	1509	ON202986.1
Uncultured Pseudomonas sp. Clone T0812 16S ribosomal RNA gene, partial sequence	uncultured Pseudomonas sp.	1149	1907	89%	0	92.95	1369	HQ616312.1
P.fluorescens 16S rRNA gene	Pseudomonas fluorescens	1149	2154	99%	0	93.82	1507	Z76662.1
Pseudomonas fluorescens strain BG20 16S ribosomal RNA gene, partial sequence	Pseudomonas fluorescens	1144	2071	96%	0	93.18	1464	KF699879.1
Pseudomonas fluorescens strain Kh22 16S ribosomal RNA gene, partial sequence	Pseudomonas fluorescens	1118	1982	94%	0	92.22	1446	MG982452.1
Pseudomonas putida strain BACD4 16S ribosomal RNA gene, partial sequence	Pseudomonas putida	1116	1990	96%	0	92.21	1498	KF413409.1
Uncultured bacterium clone UUF213 16S ribosomal RNA gene, partial sequence	uncultured bacterium	1116	2030	96%	0	92.21	1472	KC245368.1
Uncultured bacterium clone UUF160 16S ribosomal RNA gene, partial sequence	uncultured bacterium	1116	2025	96%	0	92.21	1470	KC245315.1
Uncultured bacterium clone UUF071 16S ribosomal RNA gene, partial sequence	uncultured bacterium	1116	2025	96%	0	92.21	1473	KC245226.1

### Analysis of the growth-promoting activity of Pseudomonas fluorescens BsEB-1

As shown in Table 2, *Pseudomonas fluorescens* BsEB-1 had no nitrogen-fixing activity, but did possess phosphorus-dissolving activity, as well as the activity of producing both IAA and siderophores. When inoculated on nitrogen-free solid Ashby’S Medium, *Pseudomonas fluorescens* BsEB-1 and *E. coli* DH5α (used as the negative control) could not grow, while the positive bacteria with the ability of nitrogen fixation could grow. These results indicate that *Pseudomonas fluorescens* BsEB-1 had no nitrogen-fixing activity.

**Table 2.** Results of growth-promoting activities of *Pseudomonas fluorescens* BsEB-1.

Treatment	Nitrogen fixation	Phosphate-solubilizing activity	IAA production	Siderophore production
*Pseudomonas sp.*	+	+	+	+++
*E. Coli*	-	-	-	+
BsEB-1	-	+	+	++

(-) no activity; (+) with activity; (++) higher activity

When inoculated on NBRIP solid medium, *Pseudomonas fluorescens* BsEB-1 could grow, which suggested that *Pseudomonas fluorescens* BsEB-1 was able to solubilize insoluble phosphorus. After culturing in NBRIP solid medium for 4 days, the content of soluble phosphorus in the bacterial solution reached 345.02 mg/L, which was calculated according to the linear equation of the standard curve of potassium dihydrogen phosphate (y = 173.83x + 40.297).

#### Pseudomonas fluorescens

BsEB-1 could also produce IAA and siderophores. After culturing in LB liquid medium with L-tryptophan for two days, the content of IAA reached 6.90 mg/L, as calculated by the linear equation of the standard curve of IAA (y = 7.8352x+0.2297). When it was inoculated in King’s B liquid medium for two days, bright fluorescence was generated, and the OD_400_ of the BsEB-1 supernatant was 0.515, while the OD_400_ of *Pseudomonas sp*. reached 1.227, which suggested that *Pseudomonas fluorescens* BsEB-1 could produce siderophores, but to a lesser extent than *Pseudomonas sp*.

### Colonization of Pseudomonas fluorescens BsEB-1 in the roots of Bletilla striata

#### Pseudomonas fluorescens

**Figure 2.**
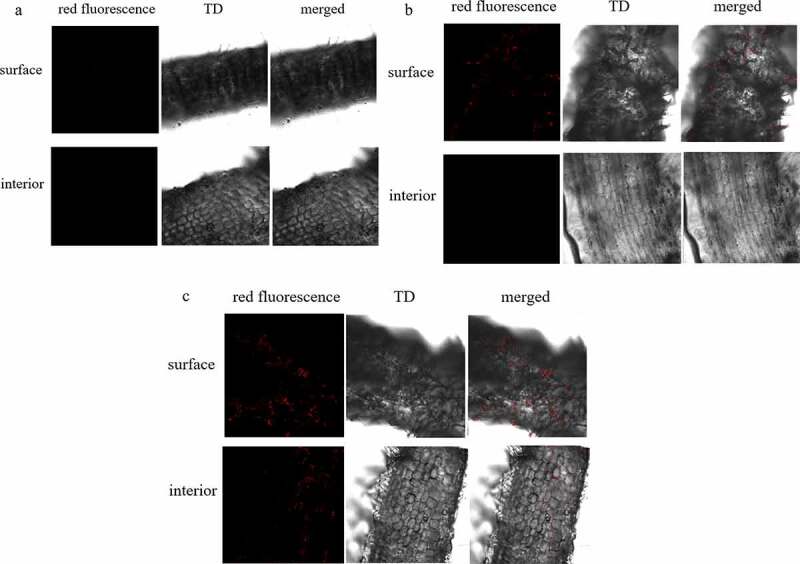
Colonization of *Pseudomonas fluorescens* BsEB-1 with red fluorescent protein in the roots of *Bletilla striata*. A: the first day after treatment with BsEB-1; B: the fourth day after treatment with BsEB-1; C: the 20th day after treatment with BsEB-1. TD: the transmitted detector image, stands for bright field.

BsEB-1 (which was transformed by pBBR1MCS-2- *mRFP1* vector) expressed mRFP1 so that it could be observed under a laser confocal microscope. After the *Bletilla striata* seedlings were co-cultured with BsEB-1 for several days, their roots were observed under a laser confocal microscope. The results showed that on day 1, BsEB-1 had attached to the root hairs of the region of maturation with weak red fluorescence, while there was no red fluorescence observed in the elongation area, meristematic area, root crown, or root interior, as shown in Figure 2a.

On the fourth day of co-culture, red fluorescence had established in the region of maturation and increased in the root hairs, but there remained no fluorescence in the interior, elongation area, or meristem area (Figure 2b). Thereafter, up until the 20th day, red fluorescence continued to appear in the root hairs and the region of maturation, but was still not found on the root surface nor within the interior of the elongation and meristem zones(Figure 2c). Throughout the whole process, there was no red fluorescence observed in the leaves or stems, nor in the roots without BsEB-1 (shown in Figure 2 and Figure 3 in the supplied files). It is, therefore, speculated that BsEB-1 only colonizes the root hairs and the region of maturation.

**Figure 3.**
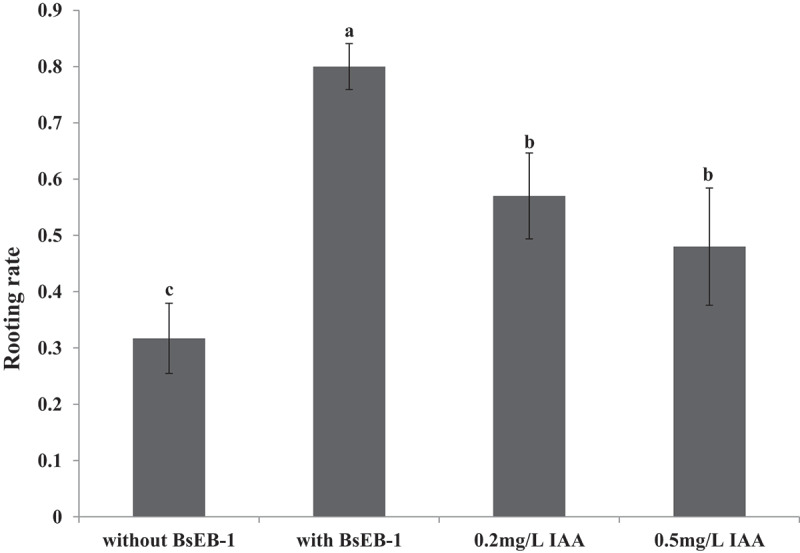
Effects of co-culturing with *Pseudomonas fluorescens* BsEB-1 or the addition of exogenous IAA on the rooting of *Bletilla striata* tissue culture seedlings. Means are values ± S.E, Means value with different letters in the figure indicates the significance difference (P˂0.05).

### Effects of co-culturing with Pseudomonas fluorescens BsEB-1 on the rooting and transplant survival rate of Bletilla striata tissue culture seedlings

The rooting ratio of *Bletilla striata* tissue culture seedlings was determined after one month of culturing (Figure 3). The rooting rate of the *Bletilla striata* tissue culture seedlings that were co-cultured with BsEB-1 (80%) was much higher than that of the *Bletilla striata* tissue culture seedlings not co-cultured with BsEB-1 (31.7%), and also significantly higher than that of the *Bletilla striata* tissue culture seedlings added with exogenous IAA (48% – 57%). The rooting rate of the *Bletilla striata* tissue culture seedlings added with exogenous IAA was also significantly higher than that of the *Bletilla striata* tissue culture seedlings not co-cultured with BsEB-1.

**Figure 4.**
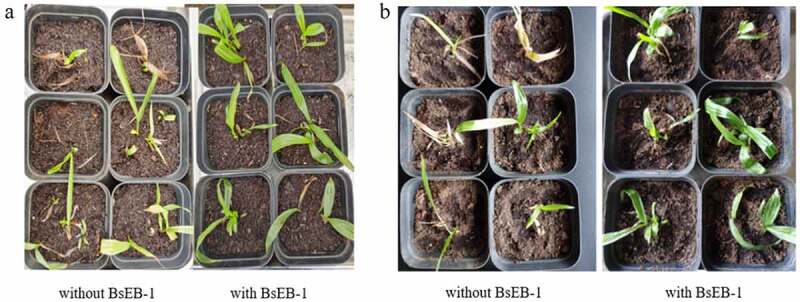
Growth status of *Bletilla striata* tissue culture seedlings after transplanting. (a) transplanted for 10 days; (b) transplanted for 30 days.

From 10 days after transplantation, it was found that the seedlings that were co-cultured with BsEB-1 grew relatively quickly, while the majority of the seedlings not co-cultured with BsEB-1 had withered, with some of these forming leaf buds again (Figure 4). After 30 days, the survival rate of the seedlings that were co-cultured with BsEB-1 was 91.67%, and the final survival rate of the seedlings not co-cultured with bacteria was only ~41.67%. The seedlings that were co-cultured with BsEB-1 also exhibited relatively better growth. These results indicated that BsEB-1 had the ability to improve the transplant survival rate of *Bletilla striata* tissue culture seedlings.

### Effect of co-culturing with Pseudomonas fluorescens BsEB-1 on the size and polysaccharide content of Bletilla striata tubers

**Figure 5.**
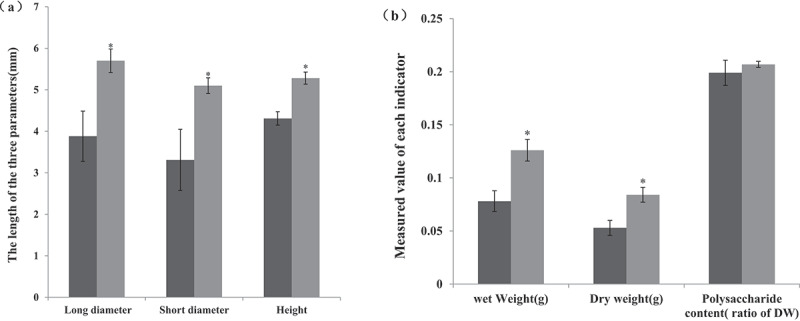
Effect of co-culture with *Pseudomonas fluorescens* BsEB-1 on the size and polysaccharide content of *Bletilla striata* tubers. (a) The size of the tuber, characterized by the long diameter, shorter diameter and height; (b) The weight and polysaccharide content of *Bletilla striata* tubers. without BsEB-1; with BsEB-1; DW: Dry weight. Means are values ± S.E, * in the figure indicates the significant difference(P˂0.05).

After having been transplanted for two months, the size and polysaccharide content of *Bletilla striata* tubers was measured. The bulbs that were co-cultured with BsEB-1 were significantly larger than those not co-cultured with BsEB-1 (Figure 5a), and the weight of the bulbs co-cultured with BsEB-1 was also significantly higher than those not co-cultured with BsEB-1, but there was no significant difference observed in either the content of *Bletilla* polysaccharide (Figure 5b), thereby indicating that *Pseudomonas fluorescens* BsEB-1 might promote the expansion of bulbs rather than the content of *Bletilla* polysaccharide.

## Discussion

The mechanisms of action employed by plant growth-promoting rhizobacteria can be divided into two categories: direct and indirect. The direct mechanism primarily refers to instances whereby some plant growth-promoting rhizobacteria synthesize or promote the synthesis of substances that improve the growth and development of the plant directly, or else change the form of the elements present in the soil so that they can be more readily absorbed by the plant; such direct mechanisms include the synthesis of auxin, 1-aminocyclopropane-1-carboxylate (ACC) deaminase, cytokinin, and gibberellin, and also include the processes of nitrogen fixation, phosphorus dissolution, and iron chelation by bacterial iron carrier.^25^ Indirect mechanisms refer to instances whereby plant growth-promoting rhizobacteria inhibit or otherwise reduce other factors from interfering with plant growth, development, or yield, for example in the case of some plant diseases,^26^ and in the presence of ACC deaminase, antibiotics, iron carriers, cell wall-degrading enzymes, hydrogen cyanide, ecological competition, induced systemic resistance, and population-quenching.^27^ In any case, the most commonly reported mechanism (which has been said to explain the positive effect of PGPR on plant growth) is the ability to produce auxin by plant growth-promoting rhizobacteria, which would be classified as a ‘direct’ mechanism. Approximately 80% of rhizosphere microorganisms have been found to synthesize and release auxin as a secondary metabolite.^28,29^

In this study, the endophytic bacterium *Pseudomonas fluorescens* BsEB-1 was isolated from the roots of *Bletilla striata*. It was found that co-culturing *Bletilla striata* with BsEB-1 could promote the rooting of tissue culture seedlings. It was also found that BsEB-1 could produce IAA, which may have contributed to the enhanced rooting. Different plants are sensitive to different levels of auxin, and the bacterial auxin absorbed by plants may change the hormone level in plants to either optimal or super-optimal.^30^ However, the simple addition of IAA did not achieve the rooting effect observed from the co-culturing of seedlings with BsEB-1, thereby indicating that other growth-promoting activities of BsEB-1 might also have played a role. One possibility could be increased phosphorus solubilization, or else BsEB-1 may have produced some other substance(s) beneficial to the rooting of *Bletilla striata* tissue culture seedlings in co-culture.

Wild populations of *Bletilla striata* in China are in danger of extinction due to excessive collection to satisfy medicinal demands. Because of this, *Bletilla striata* are now exclusively obtained by tissue culture technology and artificial planting. However, when seedlings are transferred from tissue culture conditions to either the greenhouse or field environment, their survival rates are affected. Some studies have suggested this phenomenon is mainly due to their low tolerance to environmental changes.^31^ Plant growth-promoting rhizobacteria could promote plant growth through an increased ability to fix nitrogen, thereby helping the host to obtain phosphorus and essential minerals, improving water absorption, and reducing insult from various plant pathogens.^32^ *Pseudomonas fluorescens* has been a widely studied PGPR with a variety of unique biochemical characteristics that may confer the bacteria some degree of heightened adaptability to the environment that other microbial systems are not as capable of adapting to.^33,34^ In this study, it was found that *Pseudomonas fluorescens* BsEB-1 could promote the colonization of *Bletilla striata* tissue culture seedlings, thereby indicating that BsEB-1 might enhance plant adaptability or fitness by affecting either the physiological state or by promoting new root formation.

Some studies have reported that *Pseudomonas fluorescens* could promote the accumulation of active compounds in medicinal plants. For example, some studies had found that *Pseudomonas fluorescens* could enhance both the biomass yield and the production of medicinal ajmalicine in *Catharanthus roseus* under drought-like stress.^35^ Phytochemical variations have also been observed to occur in *Salvia officinalis* inoculated with different rhizobacteria.^36^ Endophytic *Pseudomonas fluorescens* has been shown to promote the growth and accumulation of both volatile oils and sesquiterpenes in *Atractylodes lance*.^14,37^ A study found that the resident endophytes might alter the content of *Bletilla striata* polysaccharide (BSP).^23^ The polysaccharide is the plant’s primary pharmacological active component. Conversely, in the present study, the content of *Bletilla striata* polysaccharide was not found to be altered after co-culturing with *Pseudomonas fluorescens* BsEB-1. However, the roots of the tissue culture seedlings expanded significantly. The process of root expansion has been reported to be accompanied by an increase in various assimilation products, the number of cells, and the cell volume in tubers.^38^ Our results demonstrated that *Pseudomonas fluorescens* BsEB-1 could promote the growth of both seedlings and tubers of the plant *Bletilla striata*.

It has been found that plant hormones play an important role in the formation and expansion of rhizomes. The formation and development of tubers arise as the result of the synergistic effect of a variety of endogenous hormones, and this coordination between the different hormones is key to the development of tubers.^39^ The plant hormone IAA is known to promote cell enlargement and plant growth; abscisic acid exhibits an antagonistic action on the effect of gibberellin when regulating rhizome expansion;^40^ while jasmonic acid can promote the formation and development of both roots and tubers by promoting glucose metabolism.^41^ Further research on the changes of various hormones, as well as conducting transcriptomics to investigate the interaction between BsEB-1 and *Bletilla striata*, would help us to clarify the mechanism of action underlying the growth-promoting effects observed in this study.

Polysaccharides comprise the main component in *Bletilla striata*, while other chemical components, such as glycosides, bibenzyls, phenanthrenes, quinones, biphenanthrenes, dihydrophenanthrenes, anthocyanins, steroids, triterpenoids, and phenolic acids have also been shown to be important bioactive substances capable of exhibiting numerous pharmacological activities. These activities include wound healing, anti-ulcer, hemostasis, cytotoxicity, antimicrobial, anti-inflammatory, antioxidant, immunomodulation, anti-fibrotic, anti-aging, anti-allergy, and anti-itch effects.^18,42,43,44^ Whether BsEB-1 affects the production and final contents of these substances remains unknown. Further study on the metabolomics of co-cultured *Bletilla striata* will assist in understanding the effect of *Pseudomonas fluorescens* BsEB-1 on the growth of *Bletilla striata* and the synthesis and yield of its active substances.

With the constant interactions and mutual adaptation of species in nature, *Pseudomonas fluorescens* has evolved to have potential in applications involving bioremediation, pathogen control, and biofertilizing. The tendency to survive under environmental stress has made this bacterium an ideal candidate for the study of metabolic recombination in response to abiotic stresses.^45^ This characteristic has also resulted in many *Pseudomonas fluorescens* strains being used to promote plant health, such as in counteracting plant lodging in wheat and bacterial wilt in tomato.^46^ Worth acknowledging is that one strain of *Pseudomonas fluorescens* will usually possess one or more of the above characteristics, and each bacterium has its own suitable environmental and soil conditions; hence, no single organism employs all available mechanisms to promote plant growth.^47^ Joining these characterized strains, *Pseudomonas fluorescens* BsEB-1 might also be of great significance for the production and effective utilization of *Bletilla striata* and other plants.

## Supplementary Material

Supplemental Material
