# Supplementary material for: Pseudomonas fluorescens BsEB-1: an endophytic bacterium isolated from the root of Bletilla striata that can promote its growth
Source: Plant Signal Behav. 2022 Aug 3;17(1):2100626. doi: 10.1080/15592324.2022.2100626 (PMC9354766; doi:10.1080/15592324.2022.2100626)
Supplement: Supplemental Material [file KPSB_A_2100626_SM7495.docx]

SEQ. 1 The sequence of *Pseudomonas fluorescens* BsEB-1 16S rRNA

TAAGAACGCTGGCGGCAGGCCTAACACATGCAAGTCGAGCGGTAGAGAGAAGCTTGCTTC

TCTTGAGAGCGGCGGACGGGTGAGTAAAGCCTAGGAATCTGCCTGGTAGTGGGGGATAAC

GTTCGGAAACGGACGCTAATACCGCATACGTCCTACGGGAGAAAGCAGGGGACCTTCGGG

CCTTGCGCTCCCAGATGAGCCTAGGTCGGATTAGCTAGTTGGTGAGGTAATGGCTCACCA

AGGCGACGATCCGTAACTGGTCTGAGAGGATGATCAGTCACACTGGAAGTGAGACTCGGT

CCANACNCCTACGGNNGGCNGCAGTGGGGAATATTGGACAATGGGCGAAAGCCTGATCCA

GCCATGCCGCGTGTGTGAAGAAGGTCTTCGGATTGTAAAGCACTTTAMGTTGGGAGGAAG

GGCATTAACCTAATACGTTTTTTTAAAAATAGTGTTTCGACGTTACCGACAGAATAAGCA

CCGGCTAACTCTGTGCCAGCAGCCGCGGTAATACAGAGGGTGCAAGCGTTAATCGGAATT

ACTGGGCGTAAAGCGCGCGTAGGTGGTTTGTTAAGTTGGATGTGAAGGCCCCGGGCTCAA

CCTGGGAACTGCATCCAAAACTGACTGACCGCGGCGCGCGCGCTAGAGTATGGTAGAGGG

TGGTGGAATTTCCTGTGTAGYGGTGAAATGCGTTGATATAGGCGACCACCTGGACTAATA

CTGACACTGAGGTGCGAAAGCGTGGGGAGCAAACAGGATTAGATACCCTGGTAGTCCACG

CCGTAAACGATACGGTCGCAAGGTTAAAACTCAAATGAATTGACGGGGGNCCGCACAAGC

GGTGGAGCATGTGGTTTAATTCGAAGCAACGCGAAGAACCTTACCAGGCCTTGACATCCA

ATGAACTTTCTAGAGATAGATTGGTGCCTTCGGGAACATTGAGACAGGTGCTGCATGGCT

GTCGTGCGGCAAATTTCAGCTCGTGTCGTGAGATGTTGGGTTAAGTCCCGTAACGAGCGC

AACCTTGTCGATAGTTACCAGCACGTAATGGTGGGCACTCTAAGGAGACTGCCGGTGACA

AACCGGAGGAAGGTGGGGATGACGTCAAGTCATCATGGCCCTTACGGCCTGGCCTACACA

CGTGCTACAATGGTCGGTACAGAGGGGGGGCCCCAAATCGACTGCGTGAAGTCGGAATCG

CTAGTAATCGCGAATCAGAATGTCGCGGTGAATACGTTCCCGGGCCTTGTACACACCGCC

CGTCACACCATGGGAGTGGGTGCACCAGAAGTAGCTAGTCTAACCTTCGGGAGGACGGTT

ACCACGGTGTGATTCATGACTGGAGTGAAGTCGTAACAAGGTAGCCGTAGGGGAACCTGC

GGCTGGATCACCTCCGG


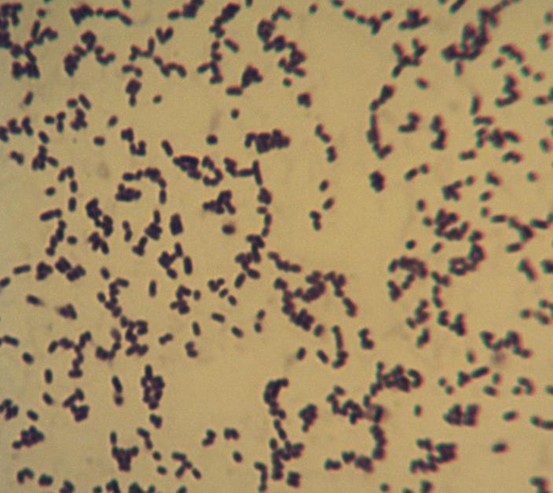


Supplied figure 1 Morphological observation of *Pseudomonas fluorescens* BsEB-1


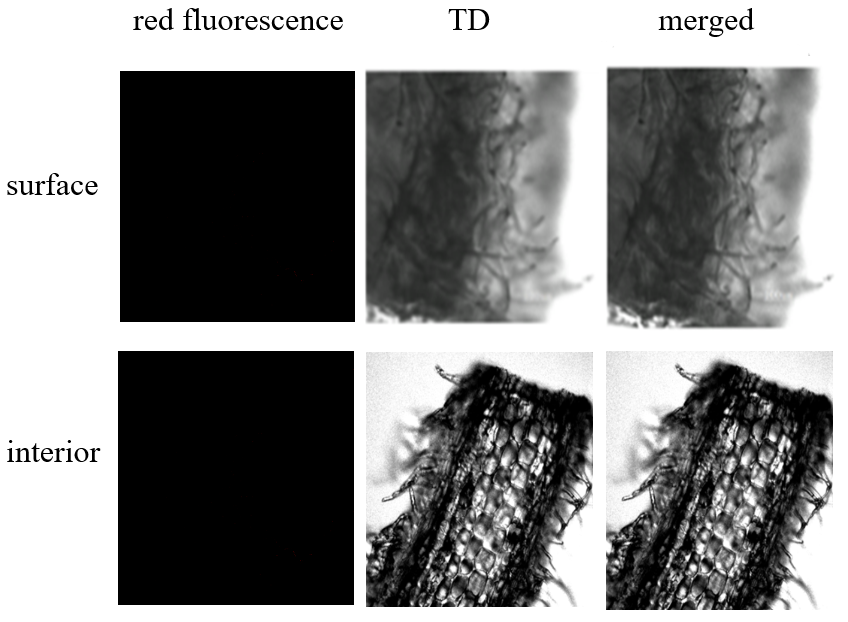


Supplied figure 2 No red fluorescence in the roots of *Bletilla striata* without BsEB-1 treatment


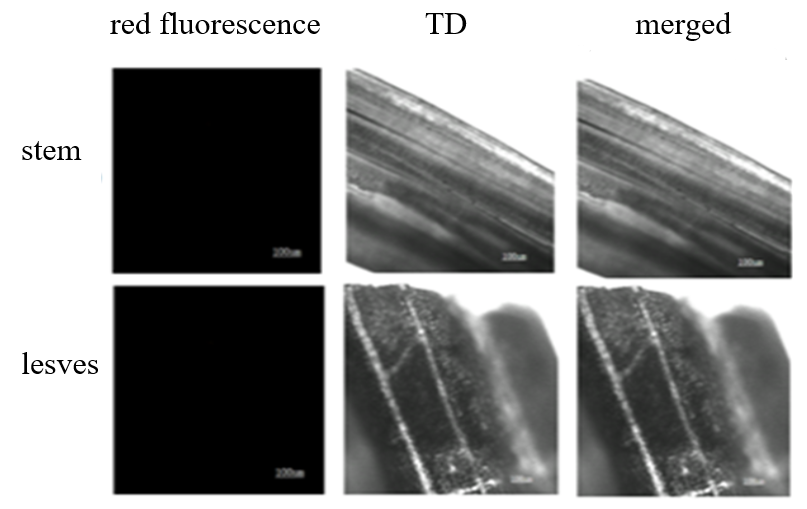


Supplied figure 3 No red fluorescence in the leaves and stems with or without BsEB-1 treatment
